# Microglia differentiation using a culture system for the expansion of mice non-adherent bone marrow stem cells

**DOI:** 10.1186/1476-9255-9-12

**Published:** 2012-04-02

**Authors:** Arnd Hinze, Alexandra Stolzing

**Affiliations:** 1 Fraunhofer Institute for Cell Therapy and Immunology, Perlickstraße 1, 04103, Leipzig, Germany

**Keywords:** Non-adherent stem cells, Bone marrow, Microglia, Differentiation

## Abstract

**Introduction:**

Studying primary adult microglia is hampered because of the difficult isolation procedure and the low cell yield. We therefore established a differentiation protocol using a culture system developed for the expansion of non-adherent bone marrow cells.

**Methods:**

Non-adherent bone marrow derived stem cells (NA-BMC) are derived by selective adhesion (‘preplating’) and are non adhesive adult stem cells. We investigated the changes in bone marrow cell populations by this repeated selective adhesion and compared the potential of the derived cells to differentiate towards microglia. Cells were differentiated with astrocyte conditioned medium (ACM) and granulocyte-monocyte colony stimulating factor (GM-CSF).

**Results:**

NA-BMC cultures show a steep raise in the fraction of stem cells during the cultivation time and the differentiation potential is of the same quality as established protocols. Around 70% of the cells are microglia defined as being positive for CD11b/CD45 and show phagocytosis activity and oxidative bursts.

**Conclusion:**

The non-adherent cell system has the advantage that is produces stem cell progenitors during expansion and provides good microglial differentiation.

## Introduction

Microglia are the immune-cells of the brain. They react to inflammatory signals, seek out and phagocytize debris, promote repair and regeneration by excreting growth factors [1]. Microglia might, however, promote neurodegenerative diseases like Alzheimer or multiple sclerosis by dysregulation and overreaction to chronic inflammatory signals. A suspected loss of their ability to function as debris clearing cells with age or an insufficient renewal of their population in age was described [1]. The role microglia play in these diseases and the possible changes the microglia population undergoes with age are the key to understand and combat the causes of neurodegeneration.

Studies of microglia *in vitro* use mostly primary microglia from mouse or rat embryos. Human microglia are difficult to obtain and they are often derived from post-mortem donors, posing some extra difficulties concerning cell viability. To study the role of microglia in neurodegeneration it is however necessary to work with adult material as the onset of the disease is age-dependent. The alternative is to use blood or bone marrow derived monocytes to derive microglia [2]. The *in vitro* generation of microglia may chart the way towards new therapeutic strategies using adult stem cells or to study the function of microglia generated from individuals of all ages and from diseased background to better understand their role in neurodegeneration.

Non-adherent bone marrow cells (NA-BMCs) harbor cells of the hematopoietic lineage [3]. NA-BMCs are known to rescue lethally irradiated mice [3,4]. Their potential to give rise to microglia could be of use in cell-based therapies of the central nervous system (CNS) [1]. Non-adherent mesenchymal stem cells (MSC) are present in NA-BMC cultures as well [3]. They give rise to fibroblastic, osteoblastic, chondrocytic and adipocytic lineages. They have been found to colonize various tissues like bone marrow, spleen, intestine, kidney and liver.

NA-BMCs might correspond to a naturally circulating population of cells [3], which carries progenitors of several somatic cell types. In line with this, cells residing in the blood have been differentiated to various cell types [5] and it is known that peripheral blood monocytes can be differentiated to microglia *in vitro*[6]. Also, fibroblast-like cells are mobilized from the bone marrow by various effects like cytokines, hypoxia and skin damage [7-10].

MSC are known to regulate microglia and other immune cells [11-14]. Bone marrow derived mesenchymal stem cells transplanted into the brain result in a reduction of amyloid-ß plaques [15] - possibly by activation of resident microglia to an amoeboid and phagocytic state. Transplanted MSC have positive effects in neurodegeneration by regulation of the microenvironment and by cell fusion [16,17]. MSC regulate activation of microglia in co-culture [18,19]. These findings suggest that inclusion of MSC in a microglia cell population might even be beneficial.

**Figure 1:**
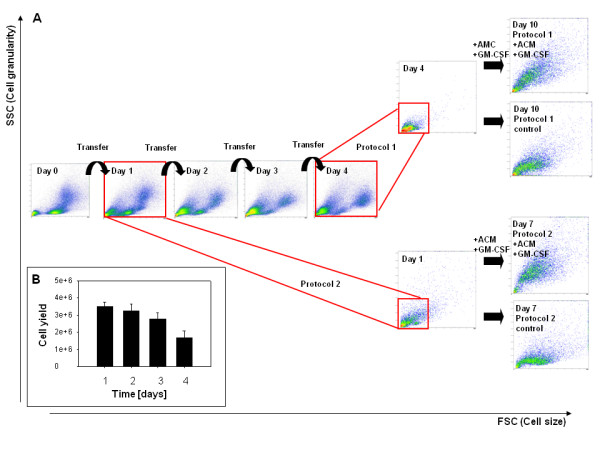
**(A) Overview showing the two differentiation methods.** Representative forward scatter (FSC) and side scatter (SSC) plots of NA-BMC are shown. **Day 0 – day 4:** Phase of selective adhesion. **Day 4 - day 10 and day 1 - day 7:** Differentiation phase. **(B)** Yield of non-adherent cells per whole bone marrow on day 1 – day 4. Repeated selective adhesion results in a falling number of cells.

To solve the problem of microglia availability and viability we explore here the improved generation of microglia cells from adult stem cells. We designed two new protocols to differentiate microglia from so called non-adherent bone marrow cells (NA-BMCs) (Figure 1), which can be expanded efficiently *in vitro* in suspension cultures without loss of stem cell properties. They correspond to a classical method for macrophage differentiation (Protocol 2) and a culture system originally developed for expansion of stem cells (Protocol 1) (Figure 1). The different stages and gradual changes during this expansion protocol are poorly investigated. The composition of these cell cultures over time, the changes in colony forming units (CFU-f) and the capacity to differentiate to microglia have not been characterized in the past. Special focus was on the functional characterization of the microglia derived by this protocol.

## Materials and methods

### Animals

Animals used for the experiments were C57BL/6 from the MEZ Leipzig and Charles River (Sulzfeld, Germany). They were handled in accordance to local animal ethics regulations.

### Bone marrow isolation and culture of NA-BMC

Femurae and tibiae of 2–3 month old C57BL/6 mice were isolated, opened and centrifuged to obtain bone marrow. 10^7^ bone marrow cells were cultivated for 24 h in a 60 mm petri dish and in 10 ml Dulbecco’s modified eagle medium (low glucose) (DMEM, Hyclone Laboratories Inc.), supplemented with 10% fetal calf serum (FCS) (Invitrogen), 10^-8^ M dexamethasone and 100 units/ml Penicillin/Streptomycin (Invitrogen). After 24 h the non-adherent cells were flushed off and transferred to a new dish (protocol 2; Figure 1). This 24 h adhesion period was repeated 4 times to derive NA-BMC cells (protocol 1; Figure 1).

### CFU-f

The non-adherent cells of day 1 (classical replating protocol to derive macrophages, protocol 2) and NA-BMCs from day 4 (protocol 1) were resuspended in 5 ml osteogenic medium (DMEM, 10% FCS, 10^-8^ M dexamethasone, 50 μg/ml ascorbic acid) in a 60 mm dish. Every 3 days, the medium was changed. After 10 days, the cells were fixed with cold ethanol and alkaline phosphatase (ALP), calcium (Alizarin red), collagen (Sirius red) and methylene blue (total colonies) staining performed. The colony numbers were determined using the program ImageJ.

### ALP staining

The cells in 60 mm petri dishes were fixed with cold ethanol for 15 min. They were washed with tap water. Tris (200 mM, pH 8.5) was mixed with naphthol phosphate ASBI (50 μg/ml) and fast red (1 mg/ml) (Fast red was always mixed fresh). 5 ml of the mixture was added to petri dishes. The dishes were shaken for 2 h at room temperature. Afterwards they were washed with tap water and allowed to dry. Photographs of the dishes were taken and colony numbers determined with ImageJ.

### Alizarin red staining (Calcium)

Cells were fixed for 15 min with ice-cold ethanol, afterwards washed with tap water. 5 ml of a solution of 1 mg/ml alizarin red in distilled water, pH 5.5 were added. The petri dishes were shaken for 2 h, afterwards washed with tap water and allowed to dry. Images were analyzed for calcium amount using ImageJ.

### Sirius red staining (Collagen)

Cells were fixed in ice-cold ethanol for 15 min and washed with tap water. 1 mg/ml sirius red was solved in picric acid. 5 ml of the mixture was added to the cells and the petri dishes shaken for 18 h at room temperature. Then the cells were washed with tap water till red color was completely eluted. The dishes were photographed and analyzed for the amount of collagen (ImageJ).

### Methylene blue staining (total colony numbers)

Cells were fixed in ice-cold ethanol for 15 min. They were washed in tap water. 1 mg/ml methylene blue was solved in 10 mM borate buffer, pH 8.8. 5 ml of the mixture was added to the petri dishes and shaken for 30 min. The dishes were washed with tap water until all dye was eluted, photographed and analyzed using ImageJ.

### Astrocyte conditioned medium

Astrocyte conditioned medium was produced by incubating medium (DMEM/10% FCS) 24 h with primary astrocyte cultures produced as described by Sievers [2].

### Differentiation towards microglia-like cells

On day 1 (protocol 2) and day 4 (protocol 1), the non-adherent cells were flushed off and transferred to a new dish. Afterwards cells were differentiated for 6 days in 10 ml DMEM/10% FCS, 50% ACM and 20 ng/ml granulocyte-monocyte colony stimulating factor (GM-CSF). Controls were cultured only in DMEM/10% FCS.

### Flow cytometry

Cell were stained for F4/80 (AF488 labelled, eBioscience) (1:250), CD11b/CD45 (AF488 and PE labelled eBioscience) (1:250 and 1:100) and CD34 (PE labelled, Caltag Laboratories) (1:100), CD45 R (RPE labelled, Southern Biotech) (1:100). Cells were trypsinized, centrifuged (300 g for 5 min) and fixed (4% paraformaldehyd). The fixed cells were washed with PBS, incubated for 2 h at 4°C with primary antibody, washed and analysed in a Beckmann Coulter FC 500 Flow cytometer.

### Phagocytosis

3*10^5^ cells were activated with 0.1 μM phorbol myristic acid for 15 min. Then they were incubated in 50 μl DMEM/10% FCS together with 50 μl 1:10 diluted opsonised beads (2.25*10^7^ beads) (Sigma) for 48 h at 37°C, 5% CO_2_. Cells were trypsinized and resuspended in DPBS (Invitrogen) and fluorescence was measured in a Beckmann Coulter FC 500.

### Oxidative burst

3*10^5^ cells were activated for 15 min with 0.1 μM PMA and controls without PMA. Activated and control cells were incubated with 50 μM DHR123 for additional 15 min. Afterwards the cells were fixed with 4% PFA and fluorescence was measured in a Beckmann Coulter FC 500. Oxidative burst was defined as signal to noise ratio (The ratio of fluorescence of activated to control cells).

### Living brain slice cultures

Brains from 2–3 month old C57BL/6 mice were transferred to cold Hank’s buffered salt solution (HBSS)/10% FCS (both Invitrogen). A VT 1000 S vibratome (Leica) was used to cut the brain in 350 μm slices. The slices were culture on a membrane (Millicell CM 0.4 μm, Millipore) at the liquid air interface of medium consisting of 50% DMEM/high glucose (HyClone Laboratories Inc.), 25% horse serum (Invitrogen), 25% HBSS (Invitrogen), 1 μg/ml insulin (Invitrogen), 100units/ml penicillin and 100 μg/ml streptomycin (Invitrogen). The brain slices were cultured for 9 days and the viability of the brain slices was assessed using DAPI/propidium iodide staining. For analysis the slices were scanned using a confocal microscope (TCS SP2, Leica Microsystems).

### Invasion of living brain tissue

On day 9 of brain slice culture, differentiated cells were treated with DIO (Invitrogen) for 20 min, washed and seeded on the top of the brain slices. A plastic ring was used to keep the cells from flowing off the slices. Cells and brain tissue were co-cultivated for additional 10 days [20]. After 10 days migration of cells into brain tissue was measured by scanning the slices with a confocal microscope (TCS SP2, Leica Microsystems) to a depth of 160 μm.

### Statistical analysis

Data is presented as means ± SE. SigmaPlot 10.0/SigmaStat 3.5 software (SYSTAT, Erkrath, Germany) was used to perform statistical analysis. For comparison of different groups ANOVA was used.

## Results

### Cell populations during selective adhesion and differentiation

The numbers of non-adherent cells in culture fell swiftly from day 0 to day 4 of selective adhesion (Figure 1B). From whole bone marrow, after 1 day of selective adhesion 3.5*10^6^ non-adherent cells remained. After 4 days of selective adhesion cell yield was 1.7*10^6^ non-adherent cells.

**Figure 2:**
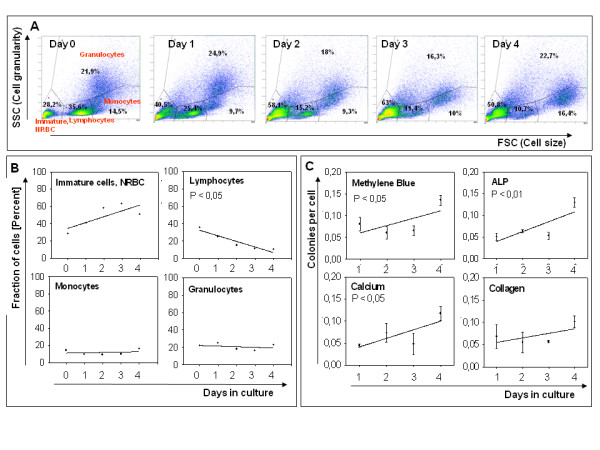
**(A) Scatter plots of NA-BMC during the selective adhesion phase.** The indicated regions define populations of immature and nucleated red blood cells, lymphocytes, monocytes and granulocytes. P is the probability of the null hypothesis of the linear regression, i. e. that the percentage fraction does not change over time. **(B)** Changes in the percentage fraction of the cell populations is shown. Lymphocyte fraction is diminished by selective adhesion. **(C)** CFU-f grown from NA-BMCs of day 1 to day 4 (n=3). Methylene blue staining was used to determine total colonies. Alizarin red (Calcium), Sirius red (Collagen) and alkaline phosphatase (ALP) staining were used to detect the capacity of NA-BMC for osteogenic differentiation.

The NA-BMC cultures were analysed at different days using flow cytometry (Figure 2A). The prominent cell populations – of immature/nucleated red blood cells, lymphocytes, monocytes and granulocytes – were defined according to their forward and side scattering (Figure 2A) as done by other groups [21]. The fraction of these populations changed during selective adhesion (Figure 2B). The fraction of lymphocytes decreased significantly from 35.6% to 10.7% during the 4 transfers of non-adherent cells (*P* ﹤ 0.05). The population of immature and nucleated red blood cells (NRBC) increased from 28.2% to 50.8% (*P* = 0.13). Monocyte and granulocyte populations did not change during the cultivation (Figure 2B). The cells derived using protocol 2 still had a prominent lymphocyte population while the cells from protocol 1 lacked lymphocytes (Figure 2A and B). The supernatant of each day was used for colony forming unit (CFU-f) assays. The frequency of methylene blue positive colonies doubled (*P* ﹤ 0.05), alkaline phosphatase (ALP) and calcium positive colonies tripled (*P* ﹤ 0.01), while collagen positive colonies did not change significantly (Figure 2C).

### Marker expression levels

**Figure 3:**
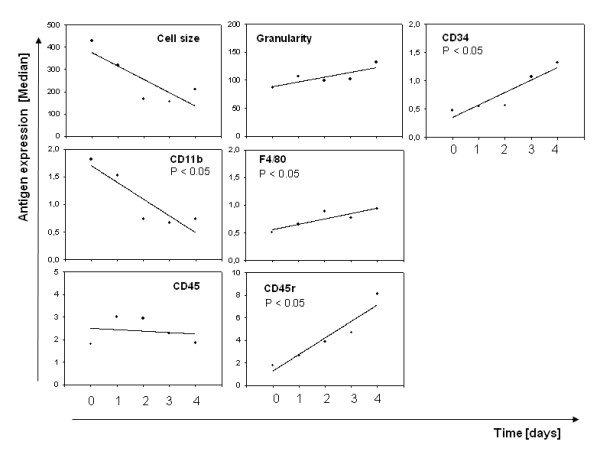
**Median of NA-BMC antigen expression during day 0 – day 4 of selective adhesion.** Both markers of differentiated (F4/80) and undifferentiated hematopoietic cells (CD34) rise. Cell size gets smaller, larger cells seem to become adherent. The regression tests for a statistically significant change over time. P is the probability of the null hypothesis – that marker expression does not change over time.

As this technique was not used before for the derivation of microglia, we wanted to describe the cultures in some detail and the changes occurring over time. During the selective adhesion period, using protocol 1, cell granularity doubled and the mean cell size decreased 3 fold (Figure 3). We were especially interested in the frequency of cells expressing macrophages/microglia markers and hematopoietic progenitor markers. The median of CD34 expression tripled significantly (*P* ﹤ 0.05), as well as 4-fold CD45 R expression (*P* ﹤ 0.05) and doubled F4/80 expression (*P* ﹤ 0.05). No change of CD45 expression was observed. CD11b expression decreased, significantly, 3-fold (*P* ﹤ 0.05) during the 4 days of selective adhesion (Figure 3).

### Microglia differentiation

The selection of non-adherent cells by adhesion was combined with differentiation media. Our new cell culture protocol (protocol 1) was compared with the classical approach (protocol 2).

It is difficult to differentiate between macrophages and activated microglia. We used a combination of non-exclusive markers and typical morphology (ramification) as specific signs of microglia (resting macrophages) as suggested by [22]. In addition we used the combination of CD11b/CD45 expression as employed by other groups [22,23] and the macrophage marker F4/80.

**Figure 4:**
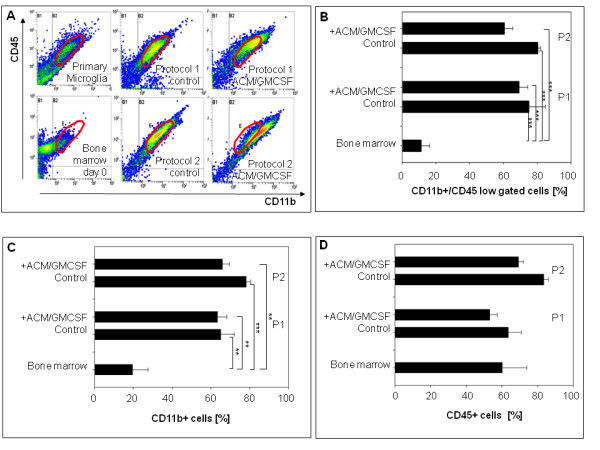
**(A) Representative scatter plots of differentiated cells.** A microglia gate was set, defined by the primary microglia population, to assess differentiation. **(B)** Percent of CD11b/CD45low (microglia gate) among the differentiated cells (n=3). Yields of cells showing the microglia markers are not different between the two protocols. A small subpopulation in fresh bone marrow (10%) shows microglia markers from the beginning. **(C)** and **(D)** Percent of CD11b^+^ and CD45^+^ cells among the differentiated cells (n=3). CD45 is already high in fresh bone marrow. P1: Protocol 1. P2: Protocol 2. *** = *P* ﹤ 0.001, ** = *P* ﹤ 0.005, * = *P* ﹤ 0.01.

**Figure 5:**
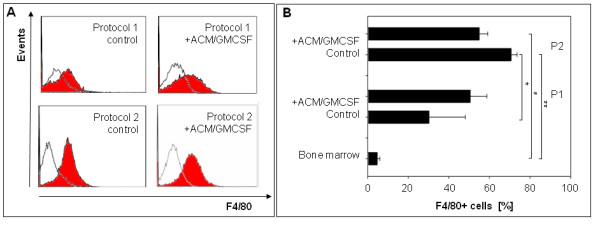
**(A) Representative histogram plots of F4/80 expression.** Gray line is the isotype control. **(B)** Percent of F4/80^+^ cells among the differentiated cells (n=3). Protocol 2 shows higher F4/80 expression than protocol 1 although, during selective adhesion, F4/80 rose steadily. P1: Protocol 1. P2: Protocol 2. *** = *P* ﹤ 0.001, ** = *P* ﹤ 0.005, * = *P* ﹤ 0.01.

The cells derived using protocol 1 and 2 were differentiated to microglia with astrocyte conditioned medium (ACM) and granulocyte-monocyte colony stimulating factor (GM-CSF) supplementation for 6 days. The cells differentiated using protocol 1 were as efficient producing microglia-like cells as protocol 2 – judged by the marker combinations suggested to be specific for microglia (Figure 4 and Figure 5).

### Functional tests

**Figure 6:**
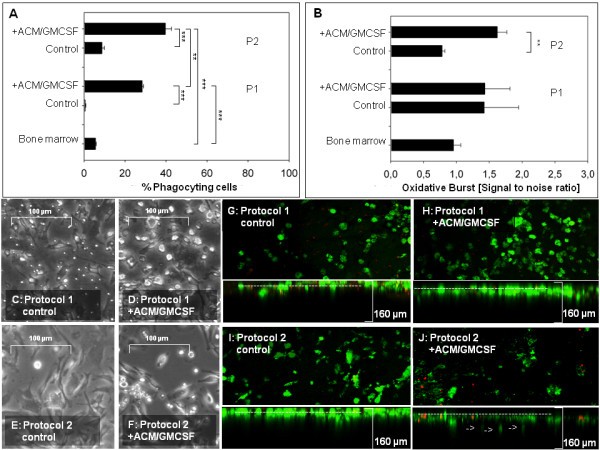
**(A) Phagocytic activity of differentiated cells.** Cells of the classical pre-plating protocol 2 show significantly higher phagocytosis of fluorescent beads. Cells un-supplemented with ACM and GM-CSF show low phagocytosis. (*n =* 3). **(B)** Oxidative burst of the differentiated cells is not different between protocol 1 and protocol 2. P1: Protocol 1. P2: Protocol 2. (*n =* 3). **(C-F)** Differentiated cells of protocol 1 are smaller and contain many non-adherent cells. Morphologies and resulting cell types are diverse. Images were taken with a Leica DMIL at 200x magnification. **(G-J)** Most differentiated cells survive in co-culture and remain largely amoeboid and round, typical for activated states. The cells were co-cultured with living brain slices. Top down and side view pictures. Differentiated cells were labeled green (DIO) and transferred onto 9 day old living brain slices. Dead cells were labeled red (propidium iodide). After 10 days of co-culture a Leica Microsystems SP2 confocal microscope was used to scan the brain slices to a depth of 160 μm (Magnification 100x). **(H, J)** Cells of the cytokine supplemented cultures invade surface up to a depth of 60 μm. *** = *P* ﹤ 0.001, ** = *P* ﹤ 0.005, * = *P* ﹤ 0.01.

Microglia derived using protocol 2 showed a significantly higher phagocytosis rate (1/4 higher) than the cells from protocol 1 (Figure 6A). Supplementation of ACM and GM-CSF lead to a significant, 4 fold increase in the phagocytic ability of the differentiated cells in both protocols. It is interesting to notice that cells of both ACM/GM-CSF supplemented and unsupplemented cultures show almost the same marker expression (Figure 4BCD, Figure 5B) but differ in phagocytosis (Figure 6A).

Microglia from cultures of protocol 1 showed the same capacity for an oxidative burst as microglia derived from protocol 2 cultures (Figure 6B). Cytokine supplementation significantly increased oxidative burst in cells derived using protocol 2 - that was present both in supplemented and unsupplemented microglia from protocol 1.

### Morphology

Cells derived from protocol 1 show many small and non-adherent cells and all differentiated cell populations show mixed morphologies as is typical for microglia cultures (Figure 6C-F). Extensive ramification was, however, not visible.

### Organotypical brain slices

The cells differentiated with protocol 2 invaded living brain tissue to a depth of up to 80 μm (Figure 6J). Most of the cytokine differentiated cells and the un-supplemented cells migrated into the surface of the slices to a depth of 30 μm.

Cells differentiated from classical derived macrophage showed amoeboid morphology (Figure 6I, J). Cells differentiated from NA-BMCs showed round morphologies (Figure 6G, H). In our qualitative migration experiments cells treated with ACM/GM-CSF or cells derived by protocol 2 migrated deepest (Figure 6J).

## Discussion

Microglia derived from adult material are difficult to obtain. Most studies have been performed on microglia from neonatal sources and cell yields from adult sources are low. In settings where cells would be used clinically, autologous sources are preferable. Bone marrow cells are routinely used for transplantation and contain different stem cell types. We have established here a protocol for the derivation of functional microglia using adult bone marrow providing a source for cell therapy or drug development. It is assumed that in the healthy brain microglia are replenished locally but that under pathological conditions and inflammation bone marrow derived cells can invade the brain and differentiate to microglia [24].

We used a stem cell cultivation method which was originally developed to expand the undifferentiated stem cell population [3,25] and tested their use as source for microglia. Repeated selective adhesion, as employed to derive non-adherent bone marrow cells (NA-BMCs), result in a rising capacity of NA-BMCs to form CFU-f. This as well as the high CD34 expression shows that the frequency of stem cells or progenitors increases during time in these cultures. The lymphocyte population in the new suspension cultures (protocol 1) gets diminished by the repeated adhesion and the fraction of immature cells increases. This corresponds to results from other groups showing in rats the increase of CFU-f initiating cells during the repeated re-plating steps [3]. The results for the monocyte lineage were mixed and we can not conclude whether, during the initial culture phase the numbers of progenitors for microglia are diminished or not.

### Microglia differentiation

No single marker distinguishes microglia from macrophages. Some publications suggest that CD45 and CD11b together might distinguish between microglia and macrophages [26]. CD45 is a marker for the hematopoietic cells and F4/80 for microglia and macrophages [27]. Our new protocol using non-adherent bone marrow cells showed the same level of differentiation towards microglial cells as protocol 2, based on the classical macrophage differentiation - judged by expression of CD11b^+^/CD45^low^ and F4/80^+^, which, together with morphology (ramification), define the microglia population as described by previous studies [22,23]. Functional microglia derived using the new protocol 1 showed slightly less phagocytosis activity than those of protocol 2, but the burst activity was of the same level.

The lower phagocytosis of cells from protocol 1 might be due to less differentiated and more primitive cells in the supernatant of day 4. It might also be an effect of the longer time in culture before differentiation. That phagocytosis is low in absence of cytokine supplementation is known [28]. Oxidative burst of the differentiated cells is not significantly higher than that of whole bone marrow. Although there are subpopulations in whole bone marrow that do show strong oxidative burst this might point to an immature microglia cell type [29].

Morphologies were mixed and only few cells showed clearly branches typical for primary resting microglia as one would expect when cells were incubated with astrocyte conditioned medium. The amoeboid and round cell morphology in the co-culture with brain slices indicates an active state in all tested protocols. This might be induced by the apoptotic cells in the slice. It is known that microglia of the brain slices migrate to the surface and phagocytize debris there [30].

## Conclusions

Repeated selective adhesion enriches for stem cells and provides great numbers of microglia, which are functionally active. This protocol provides functional adult microglia which can be used for drug development or cell therapies.

## Competing interests

The authors declare that they have no competing interests.

## Authors’ contributions

AH carried out all experiments and wrote the manuscript. AS designed & coordinated the study and contributed to writing the manuscript. All authors read and approved the final manuscript.
